# What drives cooperative breeding?

**DOI:** 10.1371/journal.pbio.2002965

**Published:** 2017-06-23

**Authors:** Walter D. Koenig

**Affiliations:** 1 Lab of Ornithology, Cornell University, Ithaca, New York, United States of America; 2 Hastings Reservation, University of California, Berkeley, California, United States of America

## Abstract

Cooperative breeding, in which more than a pair of conspecifics cooperate to raise young at a single nest or brood, is widespread among vertebrates but highly variable in its geographic distribution. Particularly vexing has been identifying the ecological correlates of this phenomenon, which has been suggested to be favored in populations inhabiting both relatively stable, productive environments and in populations living under highly variable and unpredictable conditions. Griesser et al. provide a novel approach to this problem, performing a phylogenetic analysis indicating that family living is an intermediate step between nonsocial and cooperative breeding birds. They then examine the ecological and climatic conditions associated with these different social systems, concluding that cooperative breeding emerges when family living is favored in highly productive environments, followed secondarily by selection for cooperative breeding when environmental conditions deteriorate and within-year variability increases. Combined with recent work addressing the fitness consequences of cooperative breeding, Griesser et al.’s contribution stands to move the field forward by demonstrating that the evolution of complex adaptations such as cooperative breeding may only be understood when each of the steps leading to it are identified and carefully integrated.

## Introduction

Soon after W. D. Hamilton revolutionized behavioral ecology with his ground-breaking papers formalizing the theory of inclusive fitness [1], field biologists swarmed out into the world to critically examine behavioral phenomena that were potentially dependent on genetic relatedness for their evolution. Among the more notable of these behaviors was that of cooperative breeding, in which individuals of the same species beyond a breeding pair—“helpers” or “helpers at the nest”—appear to altruistically cooperate to raise young at a single nest or brood. Once considered quite rare [2,3], cooperative breeding is now thought to characterize at least 13% of perching birds (order Passeriformes) and 9% of all bird species [4,5] as well as a smaller proportion of mammals and fishes. Although the frenzy following Hamilton’s papers has subsided, studies of cooperative breeders, which include some of the longest continuous behavioral field studies ever undertaken, continue to fascinate ecologists and provide novel insights into the interplay of competition and cooperation in animal societies [6].

What drives the evolution of cooperative breeding? One clearly important factor is Hamiltonian kin selection, although the precise role that genetic relatedness plays is to some extent controversial. In support of the hypothesis that kin selection plays a key role in providing fitness benefits to helpers, the majority of cooperative breeders are indeed composed of family groups. An intuitively pleasing extension of this hypothesis is the idea that monogamy, because of its presumed role in enhancing genetic relatedness within family groups, has been foundational to the evolution of cooperative breeding and eusociality [7]. Although supported by phylogenetic analyses in birds and mammals [8,9], a parallel analysis in cichlid fishes has failed to support this scenario [10], which also suffers from logical flaws, at least when applied to vertebrates [11,12]. These flaws include the fact that a nontrivial number of cooperative breeders are not family based [13], and those that are family based include some of the most genetically promiscuous species known [14].

Beyond the importance, both real and potential, of kinship and inclusive fitness, the one thing that almost all workers agree on is that ecological factors play a key role in driving cooperative breeding. The earliest and most widespread ecological hypothesis for cooperative breeding focuses on what are now generally known as “ecological constraints” or “habitat saturation.” Based originally on a proposal by Robert Selander to explain cooperative breeding in *Campylohynchus* wrens [15], the idea is that it is unusually difficult for offspring of such species to disperse and obtain a breeding position because the habitat is “filled up” (saturated), and thus, there are only rare opportunities for dispersal and independent breeding. Instead, young individuals facing these ecological constraints remain in their natal territory in which they “make the best of a bad job” by helping to feed younger siblings and thereby gain at least some inclusive fitness benefits. Several prominent, early studies of cooperative breeders, including those on Florida scrub-jays (*Aphelocoma coerulescens*) [16], acorn woodpeckers (*Melanerpes formicivorus*) [17], and red-cockaded woodpeckers (*Picoides borealis*) [18]—all 3 of which include “helping at the nest” by offspring living in family groups—converged on the importance of this hypothesis. The apparent generality of the hypothesis made it seem possible, at least briefly, that “…the ‘dilemma’ posed by cooperative breeding is resolved” [3].

But, alas, such optimism was short-lived. Not only were flaws in the logic of habitat saturation pointed out—including the fact that many or even most species are ecologically constrained in some way but do not delay dispersal or breed cooperatively [19,20]—but studies of other species soon demonstrated that the habitat of at least some cooperative breeders was not saturated in any meaningful sense. Equally disturbing, meta-analyses failed to support the prediction of the ecological constraints hypothesis that cooperative breeding should be found in habitats that are relatively constant and productive rather than highly variable [21–23]. Thus, ecological constraints, despite playing a key role in some species, appear to be neither necessary nor sufficient to drive cooperative breeding in general [24,25].

The field of cooperative breeding has progressed in many ways since these early studies [6], but 2 advances in particular are critical to setting the stage for the results presented by Griesser et al. in this issue [26]. The first is that there appear to be 2 nearly opposite ecological conditions that favor cooperative breeding: (1) the relatively stable, ecologically constrained, and saturated environment envisioned by the ecological constraints hypothesis and (2) the highly variable and unpredictable environments in which successful breeding is, in at least some years, difficult or impossible without the additional aid potentially provided by helpers [21,27,28]—an alternative dubbed the “hard life” hypothesis [17]. How can this apparent paradox be resolved?

One relevant observation is that both these concepts, despite being virtual opposites, involve constraints—the first on obtaining a reproductive position and the second on successful breeding once a position is obtained [27]. But this does little to resolve the problems identified above, since almost all species, cooperative breeding or not, face constraints of some sort during at least some years. Griesser et al. address this fundamental problem, which is at the heart of the question of what drives the distribution and occurrence of cooperative breeding, by making use of a second recent advance in the field.

Although early papers distinguished delayed dispersal from helping behavior, particularly when considering their fitness consequences [2,3], few thought more deeply about the potentially separate evolutionary drivers of these phenomena since they almost always seemed to go together. Then came the Siberian jay (*Perisoreus infaustus*), a species in which young typically remain in their natal territory for up to 3 years—that is, delay dispersal well into adulthood—but do not help feed at subsequent nests [29]. Siberian jays provided a unique opportunity to study the fitness consequences of delayed dispersal, independent of helping behavior—a problem that had defied others’ attempts to resolve it.

Griesser et al. [26], however, realized that the Siberian jay system offers not only a chance to better understand the fitness consequences of cooperative breeding but that it is representative of a number of noncooperative-breeding avian species in which parent–offspring associations extend beyond the period of nutritional independence [30], thus providing a fresh opportunity to investigate the evolutionary drivers of cooperative breeding. Using an impressive database on the natural history and social behavior of 3,005 terrestrial avian species, they categorize species as being nonfamily living (55% of species), family living but not cooperative breeding (31% of species), family living and cooperative breeding (13% of species), and nonkin cooperatively breeding species (1% of species). Excluding the rare last category, they proceeded to do a phylogenetic analysis investigating the evolutionary transitions between each pair of the 3 remaining categories.

They find that the best-fitting model includes transitions between all pairs of the 3 categories, but that the transition rate from nonfamily living directly to cooperative breeding is rare compared to the transition rate from nonfamily living to cooperative breeding via the intermediate stage of family living without cooperative breeding. In other words, cooperative breeders almost always evolve from family-living but noncooperative-breeding ancestors. This insight sets the stage for a multinomial analysis investigating the ecological and climatic correlates of the 3 major categories of species, thus focusing on the potential drivers of family living separate from that of cooperative breeding.

And here’s where it gets exciting. Griesser et al. find that the apparent ecological factors distinguishing nonfamily-living species from both family-living and cooperative-breeding species are generally similar, with both of the latter species tending to occur in habitats in which rainfall is greater and growing seasons are longer. Cooperative breeders, however, are more likely to be found in environments with higher within-year variability in environmental productivity compared to family-living species that are not cooperative breeders.

These results suggest a novel resolution to the conundrum of how 2 apparently contradictory environmental conditions appear to drive cooperative breeding. Relatively stable, productive conditions favor the transition from nonfamily living to the intermediate stage of family living, whereas subsequent evolution of cooperative breeding is favored when conditions subsequently deteriorate, becoming less productive and more variable. Such a scenario fits in well with the current geographic distribution of cooperative breeding, which occurs disproportionately in Australia, southern Africa, and northern South America—places that have undergone dramatic climatic changes from past geological epochs, resulting in less productive and more variable conditions that may have favored the evolution of cooperative breeding from family-living ancestors.

Although Griesser et al.’s paper is focused primarily at the level of evolutionary origins, their hypothesis also has implications for the fitness benefits associated with family living and cooperative breeding. As such, it dovetails with recent work by Shen et al. [28], which is specifically concerned with the current adaptive value of cooperative breeding. Shen et al. suggest that populations subject to habitat saturation are likely to be living in groups because of the benefits derived from group-defended resources (“resource defense benefits”) favored by spatial environmental variability, particularly in temporally stable environments, while groups that form in order to overcome difficulties in successful breeding are likely to be gaining benefits derived directly from cooperative group behaviors (“collective action benefits”) favored in temporally variable environments. These are, to a large extent, the same ecological conditions suggested by Griesser et al. to be associated with family living and cooperative breeding, respectively.

Thus, both papers are interested in the ultimate drivers of cooperative breeding and attempt to explain how very different environmental conditions appear to drive cooperative breeding but at complementary time scales and levels of analysis. It is the longer temporal scale combined with the 2-step evolutionary progression leading to cooperative breeding envisioned by Griesser et al. that allow their analysis to potentially explain the ecological and climatic factors leading to the highly heterogeneous incidence of cooperative breeding observed on a continental scale that has until now gone largely unexplained.

The ultimate goal of these, as well as other recent broad-scale investigations of social behavior [31], is to better understand social evolution in general. Determining the evolutionary drivers and ecological correlates of cooperative breeding has long been among the thornier problems in this larger process. A full synthesis has yet to be achieved; it is not yet possible to predict, based on even intimate knowledge of the ecology of a species, whether it is a cooperative breeder or not, nor do we have a particularly satisfying explanation for cases in which only 1 of 2 related species with apparently similar ecologies living in the same environment exhibit cooperative breeding [32, 33]. Furthermore, the 2-step scenario proposed by Griesser et al. emphasizes the potential importance of helpers to overcome difficult conditions in order for helping to develop from family groups, but in at least one species, the acorn woodpecker, helpers appear to provide fitness benefits primarily when conditions are good rather than poor [34]. Nonetheless, it is insights such as those of Griesser et al. that continue to move the field forward, 50 years after Hamilton set it on its modern-day trajectory, and that will one day provide a truly satisfying general theory of social evolution.
